# Statistical modeling, optimization and characterization of andrographolide loaded emulgel for its therapeutic application on skin cancer through enhancing its skin permeability

**DOI:** 10.1016/j.jsps.2024.102068

**Published:** 2024-04-12

**Authors:** N. V. L. Sirisha Mulukuri, Sujeet Kumar, Moumita Dhara, Gupta Dheeraj Rajesh, Pankaj Kumar

**Affiliations:** a Department of Pharmaceutical Chemistry, NGSM Institute of Pharmaceutical Sciences (NGSMIPS), Nitte (Deemed to be University), Mangalore 575018, India; b Nitte College of Pharmaceutical Sciences, Bangalore, India

**Keywords:** Andrographolide, Emulgels, A431, HDFcells, MTT, FITC, Design-Expert

## Abstract

Andrographolide is a natural diterpene lactone with multiple biological effects. In the present study, a total of 11 andrographolide-loaded emulgels (ANG 1- ANG 11) were prepared by emulsification and solvent evaporation method using flaxseed oil and xanthan gum in different ratios, as suggested by the Design-Expert software. A 2-factor-5-level design was employed with different responses including spreadability, extrudability, viscosity, and drug release after 1 h (h) and 24 h. Based on the Design-Expert software response, the optimized emulgel ANG 12 was formulated and evaluated. The 24 h *In-vitro* drug release was found to be 95.7 % following Higuchi kinetics. *Ex-vivo* skin retention of 784.78 ug/cm^2^ was observed during the study. MTT assay performed on Human epidermoid carcinoma (A-431) cells demonstrated cell growth arrest at G0/G1 and G2/M phase after 24 h of ANG 12 treatment (IC_50_: 11.5 µg/ml). The cellular permeability of ANG-12 was assessed by Fluorescein isothiocyanate (FITC) assay. Compared to untreated cells (0.54 % uptake) the ANG-12 treated cells had shown 87.17 % FITC permeation. The biocompatibility study performed on non-cancerous human dermal fibroblast cells (HDF cells) shows 91.54 % viability after 24 h of the treatment showing the non-toxic nature of ANG-12. Confocal imaging had shown a significant time-dependent increase in *in-vivo* cellular uptake with enhanced, progressive penetration of the emulgel into the skin. An *in-vivo* skin irritation study conducted on Swiss albino mice confirmed the safety aspects of the ANG 12. Hence, it can be concluded that nanoemulgel of andrographolide (ANG 12) could be a novel approach to treating skin cancer.

## 1 Introduction

Skin cancer has become a global concern in recent years (Urban et al., 2021). Being an interface between the body and the external environment, the skin deters the entry of harmful microbes, chemicals, and radiations inside the body (Raina et al., 2023). Prolonged radiation exposure, chemical contact, and frequent injury lead to the development of skin cancer (Watson et al., 2024). Irrespective of its etiology and taxonomy, treating skin cancer is always a daunting task (Orthaber et al., 2017). Even though skin cancer management has advanced, there are limitations and challenges associated with it. Late diagnosis, incomplete targeting, drug resistance, immunosuppression, and high cost are a few among several (Sedighi et al., 2019). Andrographolide is a diterpene lactone with multiple biological benefits (Islam et al., 2018) such as anticancer, antioxidant, antibacterial, anti-inflammatory, immunomodulatory, neuroprotective, and hepato-protective (Dai,et al., 2019) properties. It is known to produce anticancer effects through multiple mechanisms like oxidative stress reduction, cell cycle arrest, anti-inflammatory, apoptosis, activation of JNK, p38 signaling pathway, and many more (Vetvicka and Vannucci, 2021). It arrests cell cycle progression at G0/G1 phase by upregulating the cell-cycle inhibitory protein p27, downregulating CDK4, TNF-α production, and enhancing cluster of differentiation (CD) marker expression (Rajagopal et al., 2003). The andrographolide-loaded emulgel formulation for potential anticancer effects is the groundbreaking aspect of the proposed research. The present work aims to address the problems associated with poor bioavailability and the absence of an efficient bio-carrier for transdermal penetration of andrographolide, despite its notable lipophilicity with log P 2.632 ± 0.135 (Indrati et al., 2020). The present investigation will be a worthwhile approach to enhancing andrographolide delivery and patient compliance.

Emulgels are widely used for delivering hydrophobic drugs (Patel et al., 2021). The utilization of nanotechnology in dermatological formulations offers a promising platform for topical drug delivery. Nanoemulgels, which combine nanoemulsions and conventional hydrogel systems ensure controlled drug release and prolonged skin contact (Srivastava et al., 2018). The impact of Quality by Design (QbD) principles in pharmaceutical product development is pivotal for ensuring both the quality and performance of the end product (Mwangi et al., 2021). In the current study, we have explored the anti-cancer efficacy of andrographolide-loaded nanoemulgels by utilizing the QbD approach for its optimization (Hazra et al., 2021). The objective of the current work involves the formulation and optimization of nanoemulgels using design expert software and its anticancer evaluation.

## 2 Materials & methods

### 2.1 Materials

Andrographolide was procured from TCI Chemicals. Xanthan gum, gum acacia, flaxseed oil, and glycerine were acquired from Loba Chemicals. Sodium benzoate and potassium sorbate were obtained from sd fine chemicals. Triethanolamine (TEA), Ethylene diamine tetraacetate (EDTA), and dimethyl sulfoxide (DMSO) were received from Merck Life-Sciences. A431 and HDF cell lines were procured from the National Centre for Cell Sciences (NCCS) Pune. FITC reagent was received from Hi-Media, Lab. Pvt. Ltd. Mumbai, India.

### 2.2 Methods

#### 2.2.1 Compatibility studies

Fourier-transform infrared spectroscopic (FTIR) analyses were performed to investigate chemical interactions between andrographolide and the excipients used in the formulation. FTIR spectroscopy was conducted for andrographolide, excipients (flaxseed oil, xanthan gum), physical mixture, and formulated emulgels. The analysis covered the wave number ranging from 4000-400 cm^−1^, and the measurements were carried out using the KBr pressed pellet technique on Bruker-Alpha FTIR Spectrometer from Ettlingen, Germany (Corredor-Chaparro et al., 2022).

#### 2.2.2 Strategic experimental design

The current study employs the CCD to construct linear/quadratic equations for response variables through five-level two factorial trials. Utilizing Design-Expert (Version 11, State-Ease, Inc.), the optimization process was facilitated with eleven experimental runs. The optimization of drug-loaded emulgels was performed employing a five-level two-factorial trail designating xanthan gum (A) and flaxseed oil (B) concentrations as independent variables. Conversely, spreadability (Y1), extrudability (Y2), viscosity (Y3), and drug release 1 h (Y4) and 24 h (Y5) were identified as dependent variables. Following the preliminary trials, the independent variables were set at low factorial (−1), high factorial (+1), center (0), low axial (−α), and high axial (+α) levels. A design matrix was established, encompassing eleven experimental runs, including three replicates at the center point to estimate pure error for the lack of fit test (Table 1). The general polynomial equation obtained has been represented below (equation (1).

(1) $$Y\;=\;b_{0}\;+\;b_{1}A\;+\;b_{2}B\;+\;b_{3}AB\;+\;b_{4}\;A^{2}\;+\;b_{5}\;B^{2}$$

**Table 1:** Levels in experimental design with independent variables.

Independent variables	Levels				
	−α	0	−1	+1	+α
Xanthan gum (A)	2.1	5	3	7	7.8
Flaxseed Oil (B)	4.7	9	6	12	13.2

Numerical values represent factors at different levels in the central composite design.

The term Y represents different individual factors at different levels of combination; b0 is an intercept; b1 to b5 are regression coefficients, A, and B are independent variables, and terms A^2^, B^2^, and AB represent the interaction between the independent factors and the quadratic terms. The analysis of variance (ANOVA) was employed to assess the significance of the quadratic models. Examination of the model coefficients and their associated p-values were calculated and summarized in Table 1.

#### 2.2.3 Preparation of andrographolide emulgels (ANG 1- ANG 11) and its optimization (ANG 12)

Andrographolide emulgels were prepared by a two-step process (Varma et al., 2014). Initially, the nanoemulsions were prepared by solvent evaporation and sonication thereafter (Hernandez-Giottonini et al., 2020) followed by gel preparation. In brief, 0.9 g of andrographolide was sonicated in 10 ml of acetone for 10 min. Simultaneously, 0.9 g of acacia was dispersed in water (100 ml) and sonicated for 10 min. The resulting andrographolide solution was added dropwise to the acacia solution. The resulting mixture was stirred for 10 min following the evaporation of acetone. For the oil phase, a mixture of flaxseed oil (ranging from 4.7 % to 13.9 % w/w) as suggested by design expert software, and vitamin E oil was used. The aqueous phase containing andrographolide was gradually added to the oil phase. The resulting mixture was stirred and sonicated for 10 min to get nanoemulsions. 0.3 % w/w urea solution was added as a stabilizer. The gel phase was prepared by dispersing xanthan gum (2.1–7.8 % w/w) and glycerine (5 % w/w) in water. Equal quantities of the prepared gel and emulsion were mixed thoroughly to form the andrographolide-loaded emulgels. Sodium benzoate (0.2 % w/w) and potassium sorbate (0.2 % w/w) in water were used as preservatives. The optimized emulgel (ANG12) was prepared in a similar way incorporating 6.6 g of flaxseed oil and 4.6 g of xanthan gum along with other essential excipients as per QbD and CCD approach (Table 2).

**Table 2:** Formulations using central composite design approach.

	Factor 1	Factor 2
Run	A: Xanthan Gum (% w/w)	B: Oil (% w/w)
ANG 1	5	9
ANG 2	5	9
ANG 3	5	9
ANG 4	7	6
ANG 5	2.1	9
ANG 6	7	12
ANG 7	5	4.7
ANG 8	3	6
ANG 9	3	12
ANG 10	5	13.2
ANG 11	7.8	9

Coded values of each factor A& B for different formulations (ANG 1- ANG 11).

### 2.3 Physicochemical evaluation of the formulated emulgels ANG1 − ANG 11 and the optimized formulation ANG 12

#### 2.3.1 Physical examinations

The formulated emulgels were assessed for their color, odor, texture, consistency, and phase separation properties (Shukla,et al., 2019).

#### 2.3.2 pH determination

The pH of all emulgels (1 % w/w aqueous) was measured in triplicate using a digital pH meter (RPB1000, India) (Malavi et al., 2022).

#### 2.3.3 Viscosity measurement

The spindle-type Brookfield digital viscometer (LVDV-E USA) was used for the viscosity measurement. The viscosity was measured using 6 Spindles at ten revolutions per minute (RPM) (Aiyalu et al., 2016).

#### 2.3.4 Spreadability study

To assess the spreadability of the formulated emulgels, a pulley equipped with the wooden block, a pair of mirror-image glass slides, and standard weights were used. A controlled amount of one gram of the emulgel formulation was evenly applied over the glass slides, and a second glass slide was carefully positioned over it. The lower glass slide was securely attached to a stable platform, while the upper glass slide was suspended freely from a pulley bearing a load of 20 g. The determination of spreadability involved measuring the duration it took for the upper glass slide to get released from a height of 19 cm under the load (Rompicherla et al., 2022).

#### 2.3.5 Extrudability study

It is the force needed to extrude a 0.5 cm ribbon of the emulgels from a detachable lacquered aluminum tube within 10 s (Mulukuri et al., 2023).

#### 2.3.6 Drug loading studies

The drug contents of the andrographolide-loaded emulgels (ANG 1- ANG 12) were assessed as per the reference method (Ogedengbe and Kolawole, 2024). In summary, 1 g of emulgel samples expected to contain 9 mg of andrographolide were transferred to a beaker. Subsequently, 80 mL of methanol was added to the beaker, and the mixture was heated to 50 °C for 30 min. Following this, the methanolic solution of the emulgel was filtered into a 100 mL volumetric flask, and the resultant solution was adjusted to volume with methanol. The sample was then subjected to agitation in an ultrasonic bath for 5 min to ensure complete dissolution of the drug. The absorbance of the solution was measured at andrographolide maximum absorption wavelength (228 nm) using a UV–visible spectrophotometer (Hafid et al., 2015).

#### 2.3.7 In-vitro drug release study

The drug release pattern was analyzed using the Franz diffusion cell. A total of 100–150 mg emulgels equivalent to 0.9 mg of standard andrographolide were applied over a dialysis membrane (MWCO-2 kDa) of the Franz diffusion cell. A, 25 ml of freshly prepared phosphate buffer solution (PBS, pH 7.4) was introduced into the lower compartment of the cell. The setup was stirred continuously at 37 °C. At specific time intervals At specific time intervals (0, 1,2,3, 4, 5, 10, 15, 20, 24hrs), the samples (1 ml) were collected, and replaced simultaneously with fresh medium to maintain sink conditions. The samples were collected, appropriately diluted, and examined by a UV–visible spectrophotometer at 225 nm (Sooksai et al., 2019). The optimized formulation ANG12 was further studied for release kinetics, particle size analysis, *ex-vivo* permeation, biocompatibility, cytotoxicity, cell cycle analysis, skin irritability, and *in-vivo* cellular uptake.

#### 2.3.8 Study of drug release kinetics

To know the andrographolide release pattern, *in-vitro* release data were fitted to different kinetic models like zero order, first order, and Higuchi and Korsmeyer-Peppas equations (Shah et al., 2021).

#### 2.3.9 Particle size analysis

The droplet size and PDI value of optimized emulgel ANG 12 were determined using a zeta sizer (ver. 7.13) (Dhara et al., 2023).

#### 2.3.10 Ex-vivo drug permeation/retention studies

Skin permeation and retention studies were investigated using porcine ear skin (Said Dos Santos et al., 2020). The skin sample was properly cleaned and positioned between the receptor and donor compartments of Franz’s device. ANG 5, ANG 11 AND optimized emulgel ANG 12 (1 g) were placed on the skin surface (in the donor chamber) and carefully covered to avoid evaporation. Aliquots of 1 ml were withdrawn from the receptor chamber at the intervals of 1, 2, 4, 8, 16, and 24 h. The andrographolide content was analyzed by the UV spectrometric method at 225 nm (Surapaneni and Ambade, 2022).

#### 2.3.11 Cytotoxicity study

The MTT assay was conducted to assess the cytotoxicity of the ANG 12 on 200 μl suspensions of HDF and A-431 cells (Khamwut et al., 2019). The cells were seeded in 96-well plates at a density of 20,000 cells/well and allowed to grow for 24 hrs. Subsequently, concentrations of 6.25, 12.5, 25, 50, and 100 µg/ml of ANG 12 were added to each well containing 100 µl of DMEM medium. The plates were then incubated for 24 h at 37 °C under 5 % CO_2_. The plates were removed after incubation and the spent media were aspirated. MTT reagent (0.5 mg/mL of total volume) was added to each well and incubated for an additional 3 h. The reaction was terminated by adding 100 μl of dimethyl sulfoxide (DMSO) to achieve uniform dissolution in each well. The absorbance was recorded on an ELISA reader at 570 nm wavelength (Nagaraja et al., 2021).

#### 2.3.12 Cell cycle analysis

The A-431 cells (2 x 10^5^ cells/2 ml) were cultured and incubated for 24 h after treatment with reference drug (camptothecin), andrographolide, and optimized emulgel ANG 12 at their IC_50_ concentration. Propidium Iodide (PI)/RNase staining buffer solution was used as a staining agent (Ligasova et al., 2023).

#### 2.3.13 In-vitro FITC analysis

FITC analysis was performed to measure the rate of cellular uptake of optimized emulgel ANG12. In brief, the ANG 12 was tagged with FITC coded as (FG) by A431 cell lines (Al Hoque et al., 2023). Cells were cultured in a 6-well plate at a density of 2 x 10^5^ cells/2 ml and incubated at 37 °C for 24 h in CO_2_. The spent medium was aspirated, and the cells were treated with the equivalent IC_50_ concentration of the ANG12 conjugated with FITC solution (10 µg/ml) and a control in 2 ml of DMEM medium. The cells were then incubated for 0, 0.5, 1, 2, and 4 h in the dark. At the end of the incubation period, the medium was removed from all wells, and a PBS wash was given. Subsequently, the PBS was removed, and 500 μl of trypsin-EDTA solution was added, followed by incubation at 37 °C for 4 min. 2 ml of DMEM culture medium was added, and the cells were harvested into 12 x 75 mm polystyrene tubes and centrifuged for 5 min at 300 x g at 25 °C, and the supernatant was carefully decanted. A PBS wash was performed. The PBS was decanted and the cells were analyzed by flow cytometry using the 488 nm laser for excitation and detection at 535 nm (Chaharband et al., 2018).

#### 2.3.14 Skin irritation test

The skin irritation studies were conducted on Swiss albino mice (Lulekal et al., 2019) under Institutional Animal Ethics Committee (IAEC) approval number NGSMIPS/IAEC/AUG-2023/388. Briefly, 18 healthy Swiss albino mice weighing 20–25 g were selected and grouped as, Group I (Blank gel treated), Group II (andrographolide treated), and Group III (ANG 12 treated). Before 24 h of sample application, the animals were individually weighed, and the dorsal portion of their skin was depilated. Subsequently blank, andrographolide and ANG 12 were applied to the depilated area (0.5 g/6 cm^2^) of animals in their respective groups. Skin irritation was observed for 24 h after the application of the samples (Nikumbh et al., 2015).

#### 2.3.15 In-vivo skin uptake study

FITC labelled ANG 12 was applied over the dorsal skin of Swiss albino mice. The skin tissues were collected after 2, 4 and 8 h of treatment and fixed in 10 % formalin solution. Subsequently, the fixed tissue sections were mounted and examined under confocal microscope to investigate the nanoemulgel uptake by skin layers (Hoque et al. 2023).

### 2.4 6 Freeze-thaw analysis

The freeze–thaw analyses was performed to assess the stability of optimized nanoemulgel ANG 12. In brief, 2 g of sample (ANG 12) was stored at −20 °C for 24 h into a 5 ml Eppendorf tube. After 24 h, the Eppendorf tube was kept at 22 °C and thawed for 5 h. The freeze-thawing process was repeated in triplicate and analysed for any change in appearance at the end of every cycle (Mazurkeviciute et al., 2022).

## 3 Results

### 3.1 Compatibility study

**Fig. 1:**
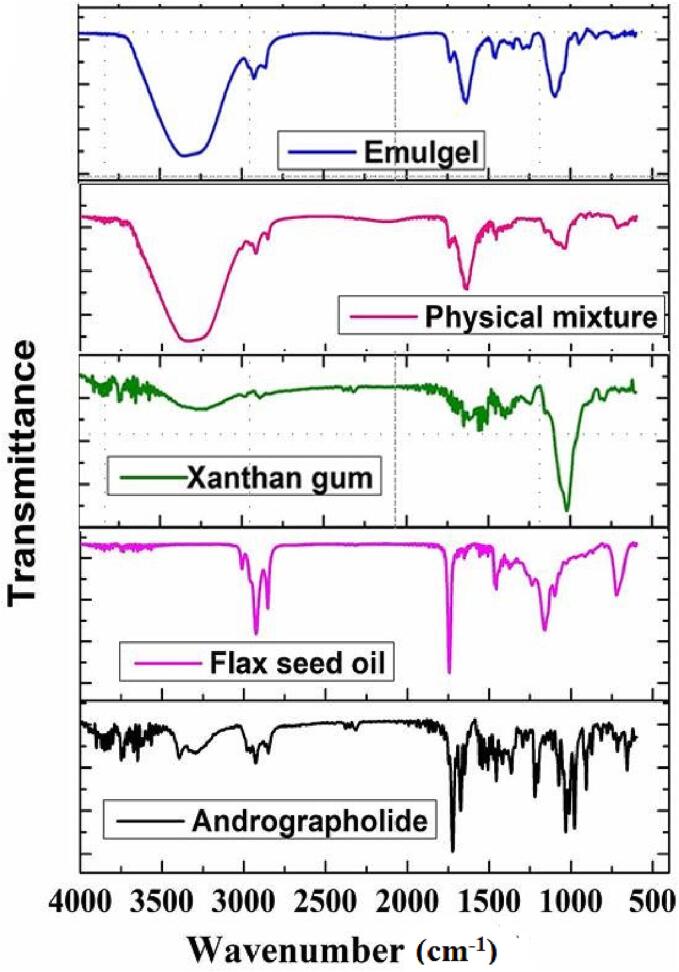
FTIR spectra of andrographolide, flaxseed oil, xanthan gum, physical mixture, and emulgel.

Chemical compatibilities of andrographolide with xanthan gum and flaxseed oil were assessed by FTIR spectroscopy. The stretching peaks observed for physical mixture and emulgel ANG12 at 3336, 3352 (–OH), 1635, 1634 (>C = O) and 1034, 1094 (−O-) cm-^1^ were found to be in close proximity to those of free andrographolide 3393 (OH), 1742 (>C = O), 1031 (−O-), xanthan gum 3277 (–OH), 1699 (>C = O), 1019 (−O-) and flaxseed oil 1742 (>C = O), 1159 (−O-) cm^−1^. The absence of any significant changes in these peak values suggests that there is no chemical interaction occurring during the formulation process, as depicted in Table 3 and Fig. 1.

**Table 3:** IR stretching values of specific functional groups.

Sample	Prominent functional groups	Stretching peak values (Wave number) (cm^−1^)
Xanthan gum	–OH	3277
	>C = O	1699
	−O-	1019

Andrographolide	–OH	3393
	>C = O	1742
	−O-	1031

Flaxseed oil	>C = O	1742
	−O-	1159
	>C = C<	1539

Physical mixture	–OH	3336
	>C = O	1635
	−O-	1034

Emulgel	–OH	3352
	>C = O	1634
	−O-	1094

Compatability studies of Andrographolide, Xanthan gum and Flaxseed oil in the emulgels to know about the interactions.

### 3.2 Optimization study by Design expert

The software for all the responses (Table 6), except for the spreadability, suggests a quadratic model, whereas a linear model was suggested for spreadability. Based on the ANOVA through model F value and p-value which states that the model terms A/ B/ AB/ A^2^/ B^2^ are significant. The obtained non-significant lack of fit was good for the model to fit in the design space (Table 4 &Table 5) Quadratic/ Linear equations were generated by Design expert software, based on the responses obtained (Table 7) for the prepared emulgels (ANG1-ANG 11). The equations (2), (3), (4), (5), (6) for all the responses are given below:

(2) $$Spreadability\;\left(Y1\right)=\;+28.75-1.32A-0.5669B$$

(3) $$Extrudability\;\left(Y2\right)=\;+29.\;93-1.85A+0.910B+0.0700\;AB-0.564\;A^{2}\;-\;4446B^{2}$$

(4) $$\mathrm{Viscosity}\;\left(Y3\right)=+5606.67+1962.58A+0.5698B-42.50AB-9.58A^{2}-42.08B^{2}$$

(5) $$\text{Drug release}\;1\;\text{h}\left(\text{Y}4\right)=+14.68-2.74\text{A}-1.28\text{B}+0.9275\text{AB}-1.46{\text{A}}^{2}-0.5348{\text{B}}^{2}$$

(6) $$24\text{hr Drug Release}\left(\text{Y}5\right)=+92.53-2.50A-0.6317B-0.1525AB-0.2996A^{2}+0.2629B^{2}$$

**Table 4:** ANOVA for Quadratic/ Linear models for spreadability and extrudability.

Factors	Spreadability (Y1)					Extrudability (Y2)					Viscosity (Y3)				
	Sum of Squares	df	Mean Square	F values	p- values	Sum of Squares	df	Mean Square	F values	p- values	Sum of Squares	df	Mean Square	F values	p- values
Model	16.49	2	8.25	7.72	0.0136	30.08	5	6. 02	30. 15	0.0010	3.083E + 0.7	5	6.166E + 06	49.47	0.0003
A	13. 92	1	13.92	13.03	0.0069	27.49	1	27.49	137.75	<0.0001	3.081E + 07	1	3.081E + 07	247.22	<0.0001
B	2.57	1	2.57	2.41	0.1594	0.2920	1	0.2920	1.46	0.2805	2. 60	1	2.60	0.000	0. 9965
AB	−	−	−	−	−	0.0196	1	0.0196	0.0982	0.7666	7225	1	7225	0.0580	0.8193
A2	−	−	−	−	−	1. 80	1	1. 80	9.02	0.0300	518.63	1	518. 63	0.0042	0.9511
B2	−	−	−	−	−	1. 12	1	1.12	5.59	0.644	10000. 98	1	10000. 98	0.0802	0.7883

**Table 5:** ANOVA for quadratic/linear models for drug release after 1 h and 24 h.

Factors	Drug release 1 h (Y4)					Drug release 24 h (Y5)				
	Sum of Squares	df	Mean Square	F values	p- values	Sum of Squares	df	Mean Square	F values	p- values
Model	88. 94	5	17. 79	25. 35	0.0015	54.71	5	10.94	199.91	<0.0001
A	60. 22	1	60. 22	85.83	0.0002	50.15	1	50.15	916.36	<0.0001
B	13. 18	1	13. 18	18. 78	0.0075	3.19	1	3.19	58.34	0.0006
AB	3.44	1	3. 44	4. 90	0.0777	0.0930	1	0.0930	1.70	0.2491
A2	12.03	1	12. 03	17. 15	0.0090	0.5068	1	0.5068	9.26	0.0287
B2	1. 62	1	1.62	2.30	0.1897	0.3904	1	0.3904	7.13	0.0443

**Table 6:** Regression analysis of Spreadability, Extrudability, Viscosity, 1 h Drug release and 24 h Drug release.

Source	Sequential p-value	Lack of Fit p-value	Adjusted R^2^	Predicted R^2^	Remarks
Model Fit Summary: Y1 (Spreadability)					
Linear	0.0138	0.1733	0.5715	0.3160	Suggested
2FI	0.5941	0.1528	0.5312	−0.2869	
Quadratic	0.3773	0.1388	0.5555	−0.4779	

Model Fit Summary: Y2 (Extrudability)					
Linear	0.0422	0.0627	0.4335	0.0318	Suggested
2FI	0.5142	0.0560	0.3934	−0.2781	
Quadratic	0.3035	0.0546	0.4729	−0.8271	

Model Fit Summary: Y3 (Viscosity)					
Linear	< 0.0001	0.0097	0.9753	0.9623	
2FI	0.8002	0.0082	0.9720	0.9588	
Quadratic	0.0282	0.0204	0.9906	0.9669	Suggested

Model Fit Summary: Y4 (1 h Drug release)					
Linear	0.0018	0.0373	0.7424	0.6030	
2FI	0.2542	0.0380	0.7588	0.4987	
Quadratic	0.0239	0.1009	0.9241	0.7428	Suggested

Model Fit Summary: Y5 (24 h Drug release)					
Linear	< 0.0001	0.0506	0.9393	0.8893	Suggested
2FI	0.9760	0.0423	0.9306	0.8011	
Quadratic	0.9120	0.0266	0.9064	0.6711	

Model fit summary suggests the type of model (Linear/ Quadratic); R^2^ determines the correlation between predicted and observed values.

**Table 7:** CCD with Factors and experimental responses (Evaluation parameters of ANG 1-ANG 11).

Run/Code	Dependent variables (Responses)^a^							
	X1 (Xanthan gum)	X2 (Flax seed oil)	Y1 (spread ability)	Y2 (Extruda bility)	Y3 (Visco sity)	Y4 (1 h release)	Y5 (24 h release)	Drug loading
	%	%	gm.cm/sec	gm/cm	poise	%	%	%
ANG 1	5	9	29	29.45	5820	14.72	92.67	88.86 %
ANG 2	5	9	30	30.12	5480	14.32	92.55	87.28 %
ANG 3	5	9	29.33	30.21	5520	15.01	92.36	87.02 %
ANG 4	7	6	26.9	26.34	7860	9.21	90.54	87.94 %
ANG 5	2.1	9	31.2	31.20	3190	15.63	95.6	86.89 %
ANG 6	7	12	28	27.23	7650	9.12	89.2	88.47 %
ANG 7	5	4.7	30.1	29.1	5450	16.32	94.23	87.08 %
ANG 8	3	6	30.1	30.6	3340	17.21	95.23	87.21 %
ANG 9	3	12	28	31.21	3300	13.41	94.5	87.74 %
ANG 10	5	13.2	27.6	29.12	5630	11.81	92.12	89.39 %
ANG 11	7.8	9	26	26.54	8020	8.8	88.5	92.03 %

(ANG 1–ANG 11) refer to emulgels prepared with 0.9% Andrographolide along with respective amounts of Xanthan gum, Flax seed oil and other excipients.

a All the values represent average of three determinations.

**Fig. 2:**
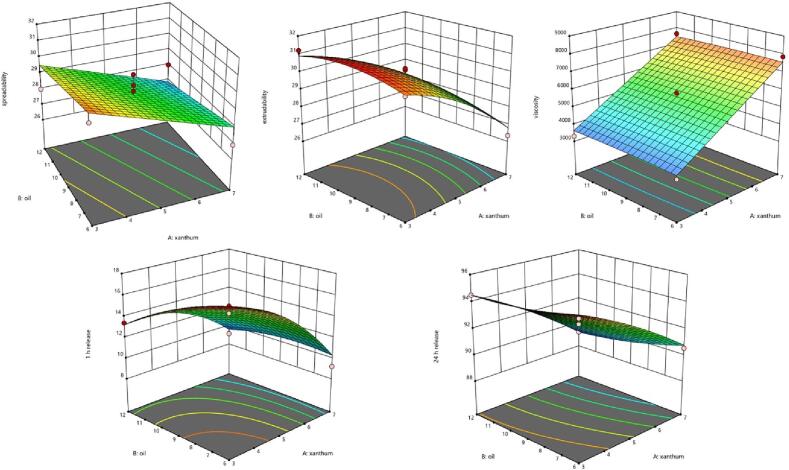
RSM plot showing the effects of xanthan gum and flaxseed oil on spreadability (Y1), extrudability (Y2), viscosity (Y3), drug release 1 h (Y4), and drug release 24 h (Y5).

In the given study, variables A and B represent the proportions of xanthan gum and flaxseed oil, respectively. A positive symbol denotes the increase in the response with higher proportions of A and B. RSM plots have clearly shown the impact of independent variables spreadability (Fig. 2). According to equation (1), an increase in xanthan gum significantly reduces spreadability, while the rise in flaxseed oil has a minimal effect. Equation (2) reveals that the concentration of xanthan gum negatively impacts extrudability, whereas higher concentrations of flaxseed oil lead to an increase in the extrudability of emulgels. Equation (3) explains the significance of terms A and B, indicating that higher proportions of xanthan gum and flaxseed oil result in elevated viscosity. Equation (4) highlights the antagonistic effect of drug release, emphasizing that an increased ratio of polymer to drug restricts drug traversal, leading to reduced drug release over a 1 h period (Table 5), suggests that the influence of flaxseed oil on the release of andrographolide may not be statistically significant. Equation (5) correlates the slow release of the drug in formulations with increased polymer content and oil proportion, suggesting that higher concentrations form a network of stiff viscous gels, impeding drug release. The model summary statistics/ regression analysis presented in (Table 6) provides valuable insights into the correlation between predicted and observed values, as quantified by the coefficient of determination (R^2^). The coefficient of determination, R^2^, is a statistical measure that indicates the proportion of the variance in the dependent variable (observed values) that can be explained by the independent variable (s) within the regression model. In the context of (Table 6), it serves as an indicator of how well the model predictions align with the actual observed values. A higher R^2^ value suggests a stronger correlation between predicted and observed values, signifying greater accuracy and precision of the model. The primary objectives in optimizing andrographolide emulgels were to achieve maximum spreadability, extrudability, minimal viscosity, and maximum drug release at both 1 h and 24 h time points. A mathematical optimization approach using the desirability function through Design-Expert statistical software was employed to determine optimal values for independent experimental variables, specifically the concentrations of xanthan gum and flaxseed oil. The design expert recommended an optimal formulation with 4.66 % xanthan gum and 6.59 % flaxseed oil. Subsequently, the formulated andrographolide-loaded optimized nanoemulgel (ANG 12) was prepared and subjected to evaluation.

### 3.3 Evaluation parameters for ANG 1-ANG 11

#### 3.3.1 Physical examination

The formulated emulgels appeared white with an agreeable odor and a smooth and uniform consistency. Importantly, there is no evidence of phase separation observed in the product.

#### 3.3.2 pH evaluation

The pH of the formulated emulgels was found between 5.9–6.3, similar to skin pH (5.4–5.9) indicating no skin irritation.

#### 3.3.3 Viscosity measurement

Viscosity is a significant parameter in semisolid dosage forms. An ideal formulation should possess minimal optimal viscosity to achieve the desired Spreadability and extrudability. The drug release was delayed in highly viscous gels, as they retain the drug (andrographolide) in the emulgel base, whereas low-viscous gels enhance both the spreadability and drug release. The viscosity of the formulated gels ranged from 2190 ± 101 cPs to 8020 ± 125 Poise (Table 7), and an increase in xanthan gum concentration resulted in increased viscosity (Pan et al., 2021).

#### 3.3.4 Spreadability study

The spreadability of an emulgel is crucial for its application. Insufficient spreadability can limit the duration of drug residence on the skin, affecting bioavailability negatively. In this study, the spreadability of all emulgels fell within the range of 25.9–30.1 gm.cm/s, signifying favorable Spreadability (Table 7).

#### 3.3.5 Extrudability study

Ensuring the appropriate consistency of an emulgel formulation is crucial for application and patient acceptance (Malavi et al., 2022). High consistency may prevent the gel from extruding effectively, while low viscosity gels may flow too quickly. In this study, the extrudability of all emulgel formulations ranged between 26.34 to 32.34 g/cm, as detailed in (Table 7).

#### 3.3.6 Drug loading studies

Andrographolide concentration in emulgel was measured by UV spectrophotometer. The andrographolide contents of andrographolide loaded emulgels (ANG 1-ANG 11) were found to be the range of 87–92 % (Table 7).

#### 3.3.7 In-vitro drug release studies

**Fig. 3:**
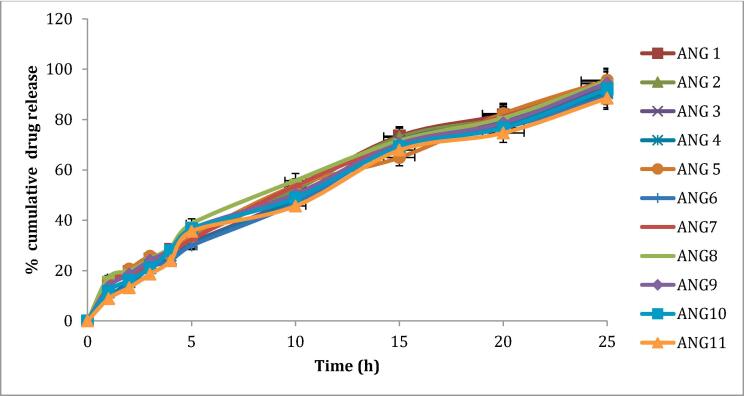
*In-vitro* drug release from emulgels ANG1-ANG11.

The *in vitro* drug release experiments were conducted using PBS (pH of 7.4) and 0.1 % w/v β-cyclodextrin (Fig. 3). Results summarized in (Table 7) revealing that all formulations exhibited controlled drug release patterns. Specifically, ANG 11 displayed a sustained drug release of 88.5 % over a period of 24 h (Fig. 3). This outcome implies that an increased concentration of polymer in the formulation leads to the slower drug release.

#### 3.3.8 Characterization of the optimized nanoemulgel (ANG 12)

**Fig. 4:**
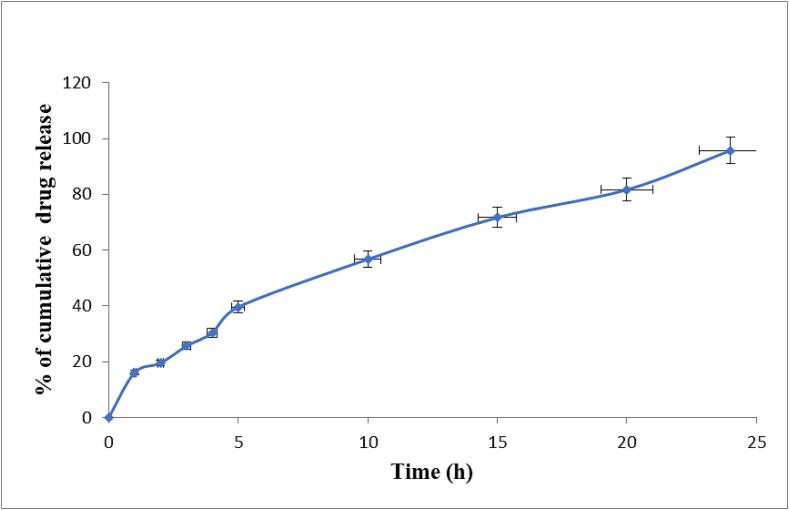
*In-vitro* drug release pattern from optimized nanoemulgel ANG 12.

The optimized nanoemulgel formulation (ANG12) is a white viscous substance with a uniform texture and glossy appearance. Various physicochemical parameters were determined, revealing the following values: pH of 6.4 ± 0.5, viscosity of 5258 ± 132 poise, Spreadability of 30.14 gm.cm/sec, and extrudability of 29.47 gm /cm. The droplet size was measured at 203 nm, with a PdI value of 0.025. *In-vitro* drug release was found to be 95.7 ± 0.5 % (Fig. 4). Andrographolide release from emulgel formulation was evaluated to follow Higuchi kinetics, based on R^2^ values, which is almost near to 1, by the fitment of the release data in various mathematic equations. (Table 8).

**Table 8:** Release kinetics of andrographolide from optimized nanoemulgel ANG 12.

*In-vitro* Drug release kinetics of ANG 12	Kinetic equation	R^2^ values
Zero order	Y = 3.5252x + 13.719	R^2^ = 0.9552
1st order kinetics	Y = 0.0465x − 2.067	R^2^ = 0.9326
Higuchi	Y = 0.0507-x0.272	R^2^ = 0.9918
Korsmeyer-Peppas	Y = 1.455x + 0.256	R^2^ = 0.7945
Hixson-Crowell	Y = 0.1077x + 0.085	R^2^ = 0.9818

#### 3.3.9 Ex-vivo permeation & skin retention study of ANG 12

**Fig. 5:**
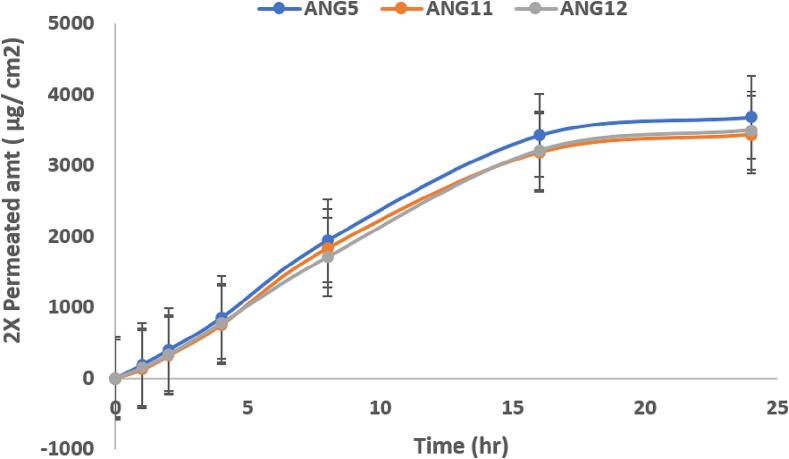
Drug permeation study through porcine skin (ANG5, ANG11 and ANG12): (2X 3880 µg ∼ 92 % of andrographolide from 1gm ANG 5; 2X 3432 µg ∼ 84 % of andrographolide from 1gm of ANG 11 and 2X 3494 µg ∼ 86 % of andrographolide from ANG12 release estimated during study.

This study aimed to investigate the permeation of the drug and its retention in the outer layers of the skin, specifically examining retention within the epidermis and dermis layers, facilitated by intercellular passages of the stratum corneum (SC). Skin permeability and penetration studies were conducted on porcine skin (Herkenne et al., 2006). Different emulgels with varying formulations can potentially influence penetration and retention by interacting with the skin layers differently, it was imperative to evaluate their performance comprehensively. Based on different characterizations and considering the release pattern, along with different physicochemical properties (Bolla et al., 2020), we selected two formulations, ANG5 and ANG11, for ex vivo permeation studies, along with the optimized formulation, ANG12. During exvivo permeation studies of ANG5, ANG 11 and ANG 12 approximately 65.87 µg, 20.34 µg and 21.11 µg of the andrographolide was deposited in the stratum corneum layer and 403.67 µg, 821.65 µg and 784.78 µg of andrographolide was retained in the dermal and epidermal layers (Table 9). The enhancement of skin permeation and retention for any topical drug delivery might be a result of drug transport mechanism across the complex skin barrier of the stratum corneum to final systemic circulation through epidermal/ dermal retention (Mukherjee et al., 2020). Prolonged retention in epidermal /dermal layer could finally justify the composition efficiency of any topical formulation in therapeutic use, especially for skin cancer (Yu et al., 2021). In Table 8 we separately showed the amount of drug deposition on stratum corneum and dermal/epidermal layer. As 1 g of ANG5, ANG11 and ANG 12 contain approx. 7.8 mg, 8.2 and 8.1 mg of andrographolides, (Referring to the drug loading from Table 7), considering these amounts 100 % we further estimated the andrographolide amount in stratum corneum/epidermal/dermal layer (Table 9.) Approximately 90 % of the drug was released within the first 24 h, while 10 % of the drug remained entrapped in the epidermal layer at 24 h. These findings highlight the superior skin permeation and retention characteristics of the optimized emulgel ANG 12 (Fig. 5).

**Table 9:** *Ex-vivo* permeation & skin retention studies: ANG5, ANG11, and ANG12.

Formulation Type	Drug content at the deposition site (µg/cm^2^)	
	Stratum corneum (SC) layer of skin (A)	Epidermis layer (without SC) + Dermis (B)
ANG5	65.87 ± 2.7	403.67 ± 45.6
ANG11	20.34 ± 4.1	821.65 ± 34.4.
ANG12	21.11 ± 3.5	784.78 ± 26.1

Andrographolide retention study in Stratum corneum (SC) layer of skin (A) and Epidermis layer (without SC) + Dermis (B) for ANG5, ANG11 and ANG12 after 24 h by porcine skin.

Considering the Supreme drug retention of ANG12 in dermal/epidermal layer it was understood that there is high possibility of drug retention at epidermal cancer site for ANG 12. Based on all physiochemical and statistical evaluation studies it was concluded that ANG 12, the optimised nanoemulgel is best suitable formula that could prove its therapeutic significance for cancer therapy.

### 3.4 Cytotoxic study

**Fig. 6:**
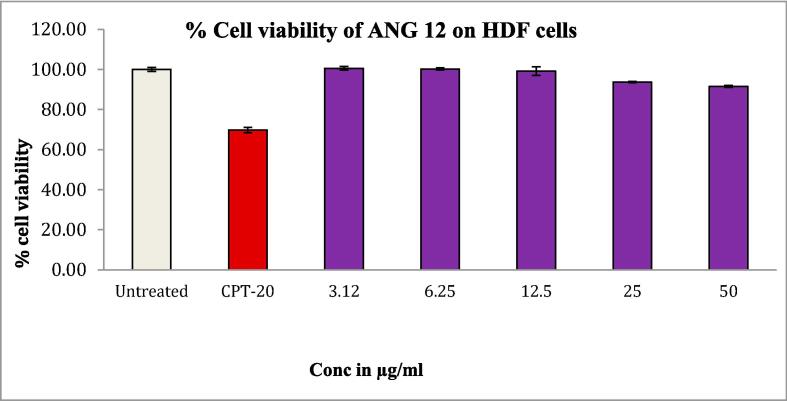
% Cell viability of HDF cells after blank, CPT-20 and ANG-12 treatment.

**Fig. 7:**
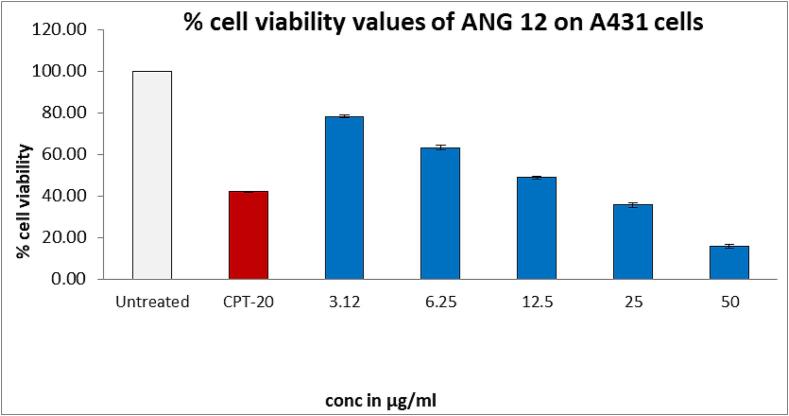
% Cell viability of A431 cells after blank, CPT-20 and ANG-12 treatment.

Biocompatibility studies were performed on HDF cells, to know the non-toxic nature of the ANG 12, before the cytotoxic evaluation against A- 431 cell lines. The cytotoxic study of ANG 12 on dermal fibroblasts HDF cell lines revealed 91.54 % cell viability after 24 h. of incubation, indicating the non-toxic nature of the ANG 12 emulgel on healthy cells (Fig. 6). On A-431 cell lines, the IC_50_ value for ANG 12 obtained was 11.51 µg/ml, which showed promising results compared to camptothecin used as a standard drug with an IC_50_ value of 20 µg as depicted in Fig. 7.

#### 3.4.1 Cell cycle analysis

**Fig. 8:**
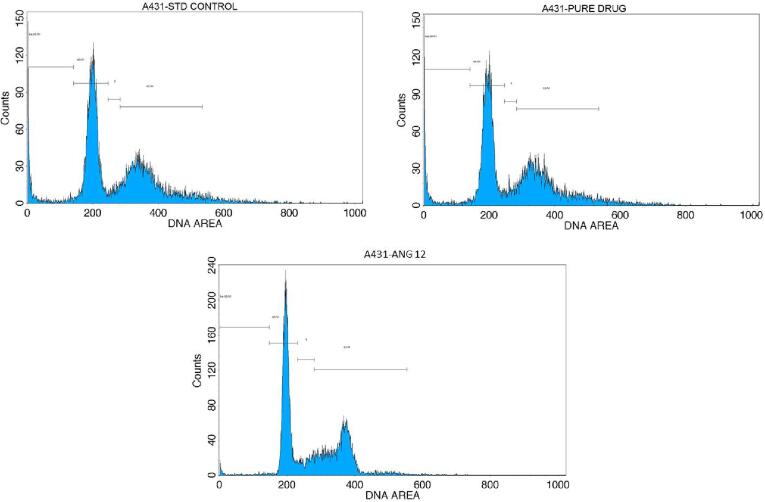
Cell cycle analysis in A-431 cells after treatment with standard drug (camptothecin), andrographolide, and optimized emulgel ANG12.

Flow cytometric analysis was done to analyze the different phases of cell cycle on treatment with standard drug (Camptothecin), andrographolide and optimized emulgel ANG12 at IC_50_ concentrations. Remarkable cell arrest by ANG 12 was observed in S phase, G2/ M phase, with 8.24 and 46.08 % growth arrest compared to camptothecin (4 % and 40.2 %) and andrographolide (3.7 %, 41.4 %) treatments (Fig. 8).

#### 3.4.2 In-vitro FITC analysis

**Fig. 9:**
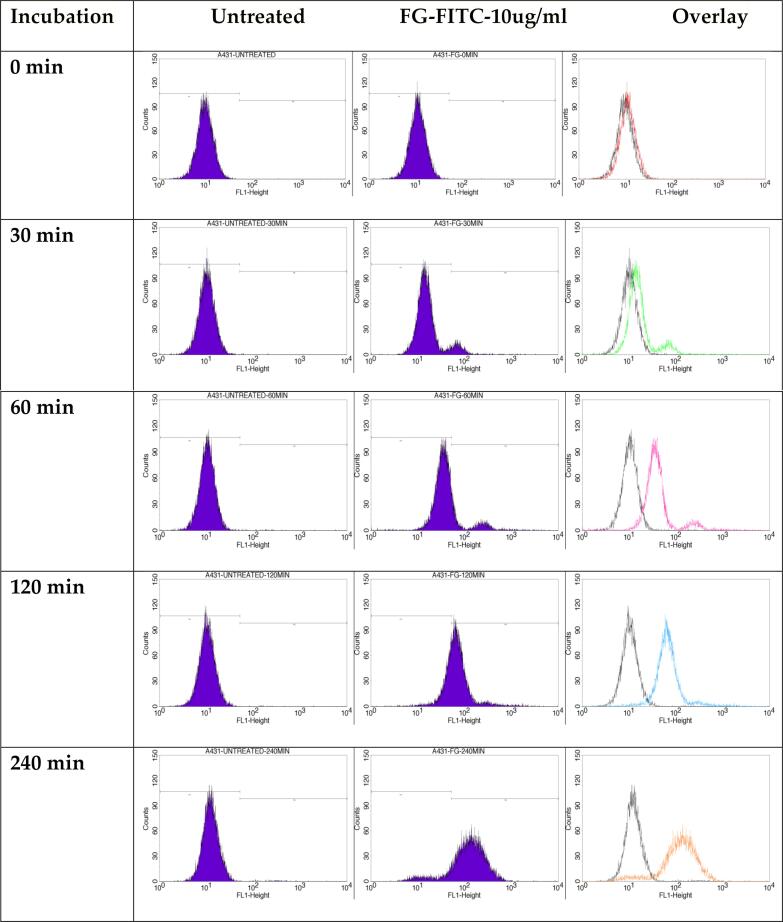
Cellular internalization study of Untreated, FG (ANG 12) formulation with 10ug/ml concentration conjugated with FITC dye against the A431 cells at different intervals.

**Fig. 10:**
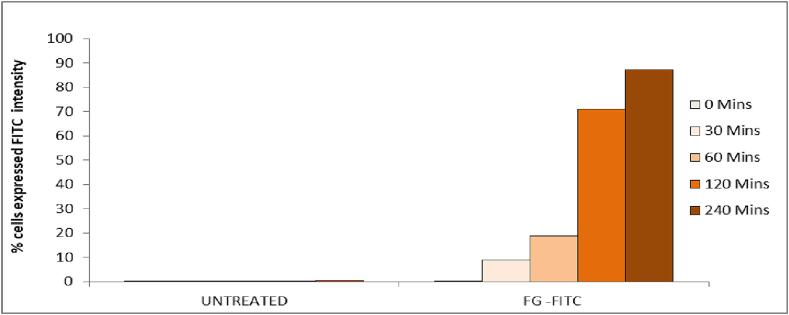
% Cells expressing FITC signal in untreated and FG-FITC (ANG 12) treated A431 cells at different intervals.

The following results were obtained from a cellular uptake study, with FITC-tagged optimized emulgel formulation (ANG 12). Fig. 9 shows the 1st lane as untreated cells and 2nd lane indicates the FG formulation conjugated with FITC and the 3rd lane indicates merged image of both untreated and FG-treated histograms.Fig. 10..

#### 3.4.3 Skin irritation study

**Fig. 11:**
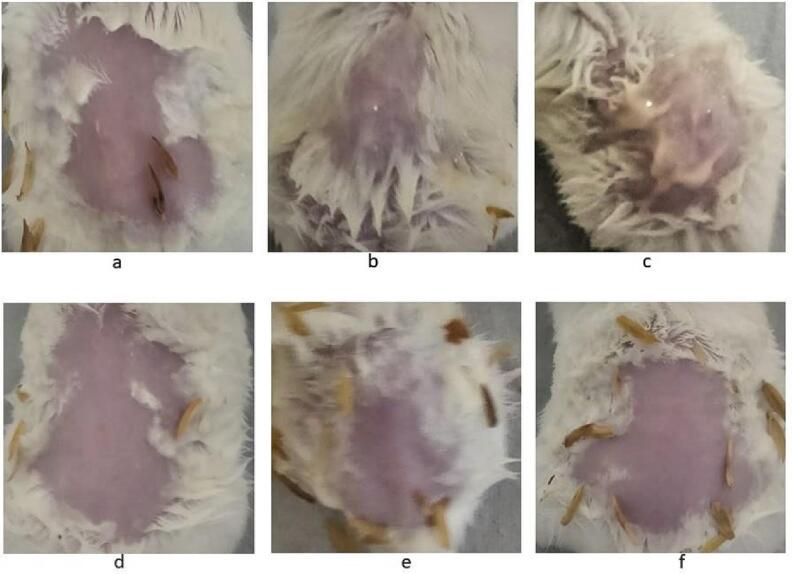
Skin irritation study; (a) Andrographolide Group I; (b) Blank gel, Group II; (c) ANG 12, Group III; Effect of skin irritation, post application of the samples on animals (d-f), respectively.

No erythema or edema formation was observed up to 24 h of blank, andrographolide, and ANG 12 treatment (Fig. 11).

#### 3.4.4 In-vivo skin uptake study

**Fig. 12:**
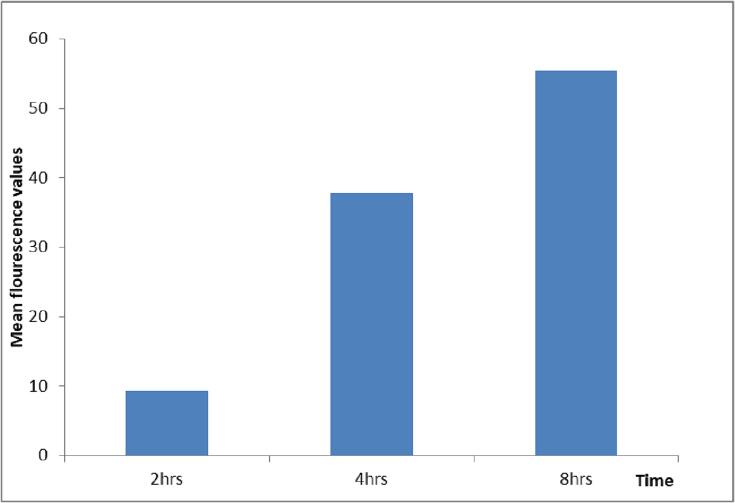
Mean fluorescence values of the ANG 12 on cell uptake study.

**Fig. 13:**
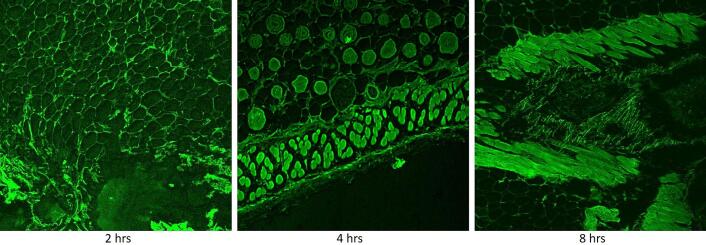
*In-vivo* cell uptake of ANG 12 by confocal microscopy.

After the topical application of nanoemulgels (ANG12) to the dorsal skin of Swiss albino mice, we examined the skin penetration of FITC-loaded emulgel (FTIC-ANG-12) under a confocal microscope. The results showed a distinct fluorescence signal in both the epidermal and dermal layers of the skin tissue, following a time-dependent pattern as depicted in Fig. 12, Fig. 13. Subsequently, we plotted the mean fluorescence uptake at different time points (2, 4, and 8 h) revealing a significant time-dependent increase in the uptake of ANG 12. These findings suggest an enhanced and progressive penetration of the emulgel into the skin over the specified time intervals.

#### 3.4.5 Freeze-thaw analysis

**Fig. 14:**
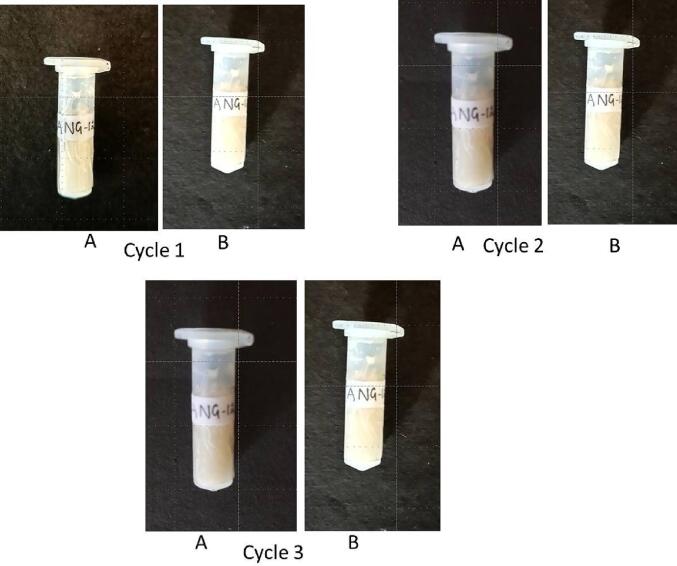
Stability testing by 3 Freeze-thaw cycles A. Frozen sample B. Thawed sample.

Emulgels (ANG 12) did not appear to change after freeze-thawing as they preserved their visual appearance. There were no signs of phase separation or macroscopic structural damage after freeze-thawing (Fig. 14).

## 4 Discussion

Andrographolide, a diterpenoid compound derived from *Andrographis paniculata*, bears anticancer properties against skin cancer. Despite the poor water solubility of Andrographolide, its logP value of 2.632 suggests potential permeability into the skin. To address the formulation challenges, andrographolide-loaded emulgels were formulated and evaluated. Polymeric agents like gum acacia and xanthan gum, known for their hydrophilic properties, are chosen for their ability to enhance drug-carrying capacity, non-irritant nature, and prolonged drug release. Furthermore, they also facilitate solubilization and partitioning of lipophilic actives into the skin layers. Xanthan gum and gum acacia acted as emulsifiers while Vitamin-E was used as an antioxidant in the formulation. The sodium benzoate and potassium sorbate were used as preservatives. Design-Expert software with CCD was used for current work to evaluate the impact of 2 factors (xanthan gum and flax seed oil concentration) on different responses during the study. The relationship between emulgel characteristics and their impact on the Andrographolide anti-cancer properties was explored. Optimizing pH levels is crucial for enhancing the stability of active pharmaceutical ingredients in emulgel formulations designed for topical applications (Shah et al., 2021). The pH of the optimized emulgel (ANG 12) was found to be 6.4 ± 0.5 indicating that the formulated emulgel is deemed safe for skin application. The main objective in optimization study through Design expert software is set to have Maximum spreadability and extrudability, which are desirable for improving patient compliance. Spreadability is an important parameter in topical formulations, as its high value facilitates easy application and increases the surface area available for drug permeation. Spreadability values above 7.5 g cm/sec show good spreadability properties (Mwangi et al., 2021). Additionally, high spreadability contributes to patient compliance by improving treatment and drug bioavailability. Formulations with low polymer concentrations are found to have better spreadability. In the case of the optimized emulgels of andrographolide ANG 12, the spreadability value of 30.14 g/cm indicates excellent spreadability, ensuring uniform delivery for enhanced skin permeation and improved retention of andrographolide against cancerous cells. The viscosity of emulgels influenced by polymer concentration and gelling capacity, plays a pivotal role in drug release. Higher concentrations of gelling agents and oils may decrease drug release due to cross-linking, reducing the polymeric structure's elasticity and restricting drug movement. Conversely, low viscosity can lead to uneven drug delivery (Samala and Sridevi, 2016). The addition of xanthan gum and oil in optimal quantities facilitates the ideal viscosity for emulgel development. In the current study, the viscosity of emulgels increased with the concentration of xanthan gum, and the optimized gel (ANG12) demonstrated a viscosity of 5258 ± 132 p. This optimized viscosity facilitated controlled drug release observed in ex-vivo studies, with maximum skin retention of 784.78 µg/cm^2^. The *in-vitro* release has shown a significant correlation with *ex-vivo* permeation studies. The optimum concentration of xanthan gum and flaxseed oil ratio provided the best release patterns. ANG 12, with its slow and constant release, attributed to the composition of xanthan gum and the interference of flax seed oil on nano-structuration, ensuring uniform drug release (Yu et al., 2021), crucial for its anti-cancer properties. Given the remarkable rheological properties and controlled drug release characteristics exhibited by the nano emulgel ANG12, our investigation delved into skin diffusion to comprehend the permeation and retention dynamics on the skin. Through a combination of *in-vivo* and *ex-vivo* experiments, a substantial amount of the drug was observed to be retained in the dermal and epidermal layers for a duration of up to 24 h. This extended retention explains the localization of andrographolide within the dermis and epidermis layers (Khan et al., 2023). The sustained accumulations and prolonged retentions of ANG12 over 24 h unequivocally affirm the superior therapeutic potential of the optimized andrographolide emulgel ANG12. To this, we observed an enhancement in the cytotoxicity threshold of andrographolide when formulated as an emulgel ANG 12, as compared to pure andrographolide. A similar trend was noted in terms of cell cycle arrest on A-431 cells, with a more pronounced inhibitory effect on cell proliferation at the S and G1/M phases for ANG 12 in contrast to both, the emulgel loaded with pure andrographolide and the Andrographolide extract-loaded gel (Mulukuri et al., 2023). Additionally, the formulated emulgel exhibited biocompatibility on HDF cells, a finding that can be correlated with the absence of skin irritation in an *in-vivo* skin irritation study conducted on Swiss albino mice. This outcome yields valuable insights into the comprehensive safety aspects of the emulgel formulation.

## 5 Conclusion

In conclusion, our research has successfully developed and optimized emulgels containing andrographolide (ANG 12) for skin cancer treatment, characterized by unique physicochemical properties and ensuring a sustained release of andrographolide. *Ex-vivo* permeation and *in-vivo* cell uptake studies have confirmed the enhanced skin permeability of the optimized ANG 12 emulgels. Additionally, ANG 12 has exhibited non-toxicity towards healthy skin cells and no skin irritation in Swiss albino mice, while demonstrating promising efficacy against skin cancer, surpassing the standard drug camptothecin. This study presents a novel phytotherapeutic strategy for skin cancer treatment, emphasizing the safety and effectiveness of the formulation. Implementation of this approach could significantly improve patient adherence.

## CRediT authorship contribution statement

**N. V. L. Sirisha Mulukuri:** Data curation, Formal analysis, Investigation, Methodology, Project administration, Writing – original draft. **Sujeet kumar:** Validation, Writing – review & editing, Data curation. **Moumita dhara:** Validation, Visualization, Data curation. **Gupta Dheeraj Rajesh:** Data curation, Formal analysis, Resources, Software. **Pankaj Kumar:** Conceptualization, Investigation, Project administration, Supervision, Validation, Writing – review & editing.

## Declaration of competing interest

The authors declare that they have no known competing financial interests or personal relationships that could have appeared to influence the work reported in this paper.
